# Retreatment of Patients With Metastatic Cutaneous Melanoma Who Relapse After Elective Checkpoint Inhibitor Discontinuation After a Complete Remission

**DOI:** 10.1093/oncolo/oyad016

**Published:** 2023-02-28

**Authors:** Kaviyon Sadrolashrafi, Wolfram Samlowski

**Affiliations:** Kirk Kerkorian School of Medicine at UNLV, Las Vegas, NV, USA; Kirk Kerkorian School of Medicine at UNLV, Las Vegas, NV, USA; Comprehensive Cancer Centers of Nevada, Las Vegas, NV, USA; University of Nevada School of Medicine, Reno, NV, USA

**Keywords:** ipilimumab, nivolumab, pembrolizumab, stereotactic ablative radiotherapy, targeted therapy

## Abstract

**Introduction:**

Checkpoint blockade has improved the response rate and survival in metastatic melanoma. Elective treatment discontinuation appears to be reasonable in most patients who have achieved a confirmed complete remission. It seems crucial to understand whether restarting immune checkpoint inhibitor therapy can induce additional responses or remissions in rare patients who relapse.

**Methods:**

A retrospective analysis identified only 10 patients who relapsed after elective treatment discontinuation after a radiologically confirmed remission. These patients were retreated with single-agent PD-1 or combined CTLA-4 plus PD-1-directed monoclonal antibodies.

**Results:**

We found an initial complete response rate of 20% (2 patients) following retreatment. With a median follow-up of 26 months, the addition of individualized salvage therapies converted an additional 4 patients to a 2nd remission. All 6 of these patients have again discontinued therapy. Three patients have died of metastatic melanoma, while another is receiving salvage therapy. Six of our 10 patients experienced grades 2-3 retreatment-related toxicity. There were no hospitalizations or fatalities.

**Discussion:**

Retreatment of relapsing patients resulted in 20% complete responses with checkpoint inhibitors. The planned addition of other treatment modalities converted another 4 patients (40%) to a durable 2nd remission. This sequential approach merits further exploration in prospective clinical trials.

## Teaser

Elective treatment discontinuation appears to be reasonable in most patients who have achieved a confirmed complete remission. This article reports on whether restarting immune checkpoint inhibitor therapy can induce additional responses or remissions in rare patients who relapse.

**Implications for Practice**

If patients with metastatic melanoma achieve a confirmed complete remission with checkpoint inhibitor immunotherapy, elective treatment discontinuation can be considered. While most complete responses appear durable, a small percentage of patients (8%-10%) eventually will relapse. Limited information is available regarding patient responses to CKI re-induction. We present a small case series demonstrating that 20% achieved another complete remission with CKI retreatment. Individualized addition of surgery, radiosurgery, or targeted agents to CKI treatment was able to convert another 4 patients (40%) to durable remissions. This sequential approach may provide a valuable option for this increasing patient population.

## Introduction

Recent advances in cancer immunotherapy have improved the rate of complete remission and survival in patients diagnosed with metastatic cutaneous melanoma. For example, in phase III clinical trial (CheckMate-067), an increased complete response (CR) rate was observed using the combination of ipilimumab and nivolumab versus nivolumab monotherapy, or ipilimumab monotherapy (22% vs. 19%, vs. 6%, respectively). With a 6.5-year median follow-up, 5-year ­progression-free survival (PFS) was 34%, 29%, and 7%, respectively, resulting in 5-year overall survival (OS) rates of 49%, 42%, and 23%.^1^ Many of the objective responses appeared to be durable.^1^ In a separate clinical trial (KEYNOTE-001), pembrolizumab, another PD-1-directed monoclonal antibody, resulted in a 16% long-term CR rate at 5 years.^2^ This therapy resulted in 5-year PFS and OS rates of 21% and 34%, respectively.

Some previous clinical trials of checkpoint inhibitor (CKI) therapy in metastatic melanoma patients were designed to require a finite period of treatment (eg, 2 years). Other clinical trials suggested continual treatment unless excessive toxicity occurred or patients/physicians decided to end treatment.^2,3^ Due to this variability in clinical trial designs, it is unknown how long patients should be treated with CKIs once they achieve remission, or whether treatment can ever safely be discontinued. In previous clinical trials, PFS appeared to show an inflection point after 1.5-2 years, with a subsequent marked decrease in the rate of melanoma recurrence (to less than 1%-2% per year). Patients who achieved a radiologic CR appeared to have the lowest risk of recurrence.^1^ This observation raised the possibility that patients who achieve CR could safely discontinue CKI treatment.

We and others have begun to evaluate the outcome of treatment discontinuation in patients who have achieved complete remission.^4,5^ In these publications, treatment was electively discontinued when patients achieved a confirmed CR documented on 2 sequential computed tomography (CT) or positron emission tomography (PET)/CT scans 3 months apart. Using this strategy, most individuals who discontinued CKI therapy remained in long-term, durable remission. Only 8.7%-10% of patients eventually relapsed, with a median time to progression over 2 years.^4,5^ Another factor that may affect the decision to electively discontinue treatment is a better understanding of the outcomes of retreatment in patients who progress after elective treatment discontinuation.

We describe our experience with CKI re-induction in a series of patients with unresectable or metastatic cutaneous melanoma who were initially treated into complete remission with CKI therapy. These patients subsequently electively discontinued therapy. We identified and evaluated the rare patients who relapsed and retreated.

## Materials and Methods

### Patient Identification

Patients for this analysis were identified by searching a Health Insurance Portability and Accountability Act - compliant electronic patient care database (iKnowMed, McKesson, Houston, TX, US) for ipilimumab, nivolumab, and pembrolizumab administration in metastatic cutaneous melanoma patients (W.S.). Each patient’s data were extracted from records (K.S.) and individually reviewed by K.S. and W.S.. These patients were treated between January 1, 2008 and December 31, 2021.

We accessed the individual medical records of each patient meeting eligibility requirements and then entered their data onto a password-protected Excel spreadsheet (Microsoft Corporation, Redmond, WA, US). Extracted data included demographic data, treatment-related data, and clinical response assessments. Treatment-related toxicities were also recorded. The addition of targeted therapeutic agents, radiotherapy, or surgery used for subsequent salvage therapy was documented. Data were de-identified after extraction before analysis. The Western Institutional Review Board (IRB) chair formally reviewed this study design and deemed it exempt from full IRB review.

Patients who discontinued initial CKI therapy because of unmanageable toxicity, failed to achieve initial remission, or died during initial CKI treatment were excluded from the analysis. Patients who relapsed after adjuvant CKI therapy for surgically resected stage II and III disease were also excluded.

### Initial CKI Treatment That Induced a Complete Remission

Each patient had received treatment with monoclonal antibodies against CTLA-4 (ipilimumab) or PD-1 (nivolumab or pembrolizumab) employing established regimens available at that time.^2,3^ In our study cohort, toxicity assessments (including appropriate laboratory tests) were performed at each treatment visit. Toxicity was graded using the National Cancer Institute Common Terminology Criteria of Adverse Events, Version 4.0.^6^ Treatment was delayed if significant toxicity (ie, grades 2-4) occurred. Glucocorticosteroids or other immunosuppressants were administered if required.^7^ Patients were cautiously restarted on original treatment regimens once toxicity subsided to grade 1 or less. Dose reductions were not performed.

We confirmed the duration of the initial complete remission (R1) based on radiologic assessments according to version 1.1 of the response evaluation criteria in solid tumors (RECIST v1.1) guidelines.^8^

### Monitoring After Initial CKI Induced Complete Remission

Patients who achieved an initial or subsequent complete remission were monitored by clinical examination and laboratory testing every 3 months for the first 2 years, every 6 months between years 2 and 5, and annually after 5 years. CT or PET/CT scans were performed every 6 months during the initial monitoring interval and then annually after 2 years. Clinical and radiologic reports that showed disease progression were used to calculate the initial PFS-1.

### CKI Retreatment After Relapse

CKI re-induction occurred upon radiologic or biopsy confirmation of recurrent disease. All relapsing patients were considered for enrollment into clinical trials at relapse. If ineligible for clinical trials, patients were retreated with the most effective CKI therapy available. Five patients (50%) were retreated with ipilimumab and nivolumab combination therapy using 1 of 2 equivalent published regimens.^9^ The remainder received PD-1 monotherapy (with either pembrolizumab or nivolumab) prior to the approval of the combined ipilimumab-nivolumab regimen.^2,3^ We monitored the retreatment response (R2) with sequential radiographs as described. In addition to CKI therapy, all patients who progressed with oligometastatic disease were considered for individualized therapy, such as surgical resection, or disease ablation using stereotactic ablative radiotherapy, in addition to ongoing PD-1 maintenance therapy. If patients had a targetable mutation (eg, BRAF V600E) and did not respond to CKI retreatment, cautious addition of BRAF ± MEK inhibitors to PD-1 therapy was considered.^10,11^ Data collection concluded on October 30, 2022.

### Statistical Analyses

Descriptive statistics were calculated via an Excel spreadsheet (Redmond, WA, US), including median, range, and standard deviation. A Kaplan-Meier analysis evaluated PFS and OS following retreatment.^12^ PFS was calculated for induction therapy from the beginning of initial CKI treatment until the date of documented relapse (R1). Secondary PFS (R2) was calculated from the retreatment start date until the most recent clinic visit (if alive) or date of progression based on scans. OS was calculated from the start of retreatment until the date of death or the date of the last clinic visit (if alive).

## Results

### Individual Patient Characteristics

By searching medical records dating back to 2011, 540 patients were treated with CKI therapy for metastatic or unresectable melanoma, with 190 achieving complete remission (35.2%). These patients electively discontinued therapy after confirmatory radiographs. Only 10 patients were identified, who relapsed (1.9%). These 10 patients were subsequently retreated with CKI therapy at relapse.

Patient characteristics are shown in Table 1. All patients in this series were Caucasian. Five patients (50%) were male and 5 (50%) were female. The median age of the entire sample at the time of retreatment was 56.5 years, with a range of 26-70 years. Tumors in 3 patients (30%) expressed a BRAF V600E mutation, while 1 contained an internal rearrangement of exon 8 of BRAF. Three patients had NRAS mutations (2 Q61R and 1 Q61K). Two patients did not have targetable mutations in BRAF, NRAS, NF1, or c-KIT (quadruple negative). The mutational status of 1 patient was unknown. All patients had distant metastases of their melanoma at the time of recurrence.

**Table 1.** Individual patient characteristics and re-treatment outcomes.

UPN	Age	Sex	Mutation	CKI-1	Doses CKI-1	PFS-1 (mo)	Site of relapse	CKI-2	Doses CKI-2	TT added	Other therapy	LDH at relapse	OR-2	PFS-2 (months)	OS (months)	CKI-2 toxicity	Status
1	60	F	WT	I	12*	67.7	Disseminated non-CNS	I	4	–	LND	Normal	CR	104.3	173.2	Diarrhea	NED
2	53	M	BRAF: V600E	I	4	95.2	Disseminated non-CNS	I+N	4	E+B	–	Elevated	CR	27.1	122.8	Diarrhea	NED
3	70	F	BRAF: Internal rearrangement	I+N	10	16.7	Disseminated non-CNS	I+N	3	D+T	–	Normal	PR	1.4	18.1	None	DOD
4	50	M	NRAS: Q61K	I+N	10	20.3	Solitary non-CNS	I+N	2	–	LND	Normal	CR	17.8	38.5	None	NED
5	63	F	NRAS: Q61R	I+N	6	8.8	Disseminated non-CNS	I+N	2	–	–	Normal	PD	3.0	11.9	Rash, pruritus, vertigo	AWD
6	49	F	WT	I	4	96.0	Solitary non-CNS	I+N	3	A	–	Elevated	PD	23.0	119.2	Rash, diarrhea, fever	AWD
7	68	M	NRAS: Q61R	I	10*	31.4	Unresectable local disease	P	11	–	SART	Normal	CR	68.3	100.3	Diarrhea	NED
8	37	M	BRAF: V600E	N	12	29.8	Disseminated non-CNS and CNS	N	1	E+B	WBRT	Elevated	PD	1.1	35.3	Nausea, vomiting	DOD
9	26	M	BRAF: V600E	I+N	7	5.7	Unresectable local disease	N	6	E	–	Normal	CR	41.3	47.1	None	NED
10	67	F	Unk	I	7*	14.4	Unresectable local disease	P	11	–	SART	Elevated	CR	9.4	28.4	None	NED

*Ipilimumab maintenance doses received every 3 months.

Abbreviations: A, everolimus; AWD, alive with disease; B, binimetinib; CKI-1, initial checkpoint inhibitor induction regimen; CKI-2, checkpoint inhibitor re-induction agent; CKI-2 (retreatment regimen; CNS, central nervous system; CR, complete response; D, dabrafenib; DOD, death from disease; E, encorafenib; F, female; I, ipilimumab; LDH, lactate dehydrogenase; LND, lymph node dissection; M, male; N, nivolumab; NED, no evidence of disease; OR-2, objective response to CKI-2 treatment; OS, overall survival from beginning of initial CKI induction agent (CKI-1) treatment to date of last clinic visit or death; P, pembrolizumab; PD, progressive disease; PFS-1, progression-free survival from beginning of CKI-1 treatment to disease progression; PFS-2, progression-free survival from beginning of CKI-2 treatment to disease progression or last clinic visit; PR, partial response; T, trametinib; TT, targeted therapy; SART, stereotactic ablative radiotherapy; Unk, unknown mutational status; UPN, unique patient number; WBRT, whole brain radiotherapy; WT, wild-type (no BRAF, NRAS, NF1 or KIT mutations).

All patients initially were treatment naïve prior to initial CKI therapy for metastatic melanoma. During their initial CKI induction, 5 patients (50%) had received ipilimumab as monotherapy. Three of these patients received 4 induction doses and subsequent maintenance therapy with ipilimumab every 3 months in clinical trials. Two additional patients achieved complete remission after 4 induction doses of ipilimumab and did not receive maintenance therapy. Four of the ipilimumab-treated patients received concomitant stereotactic radiosurgery (SRS) in addition to CKI therapy to ablate oligometastatic disease in a clinical trial. The other 5 patients were treated with a variety of regimens: 1 (10%) received nivolumab as monotherapy, and 4 (40%) received ipilimumab and nivolumab as combination therapy. All 10 patients achieved a radiographically confirmed complete remission as their best overall response to initial CKI induction prior to treatment discontinuation. The median PFS from initial CKI induction to initial disease progression (R1) was 25 months (±34.7 months SD); this ranged from a minimum of 5.7 months to a maximum of 96 months prior to documented relapse. Six patients (60%) had grades 1-2 toxicity related to their initial CKI treatment, including fever, chills, rash, pruritus, colitis, dyspnea, and vitiligo. Treatment discontinuation was considered after 2 sequential scans documenting a radiographic remission as previously described.^4^

### CKI Retreatment Outcomes

The choice of salvage therapy was based on the most effective treatment available at the time of relapse. This included single-agent PD-1 therapy (pembrolizumab or nivolumab in 5 patients) or combination therapy (with ipilimumab and nivolumab in 5 patients) (Table 1). One patient initially treated with ipilimumab was reinduced with single-agent ipilimumab, 2 ipilimumab-treated patients were re-induced with pembrolizumab, 2 ipilimumab-treated patients were reinduced with ipilimumab + nivolumab. One nivolumab patient underwent re-induction with nivolumab. Four patients initially received ipilimumab + nivolumab combination therapy. Three underwent reinduction with ipilimumab + nivolumab. The 4th patient had significant toxicity with the combination regimen and was re-induced with single-agent nivolumab.

**Figure 1.**
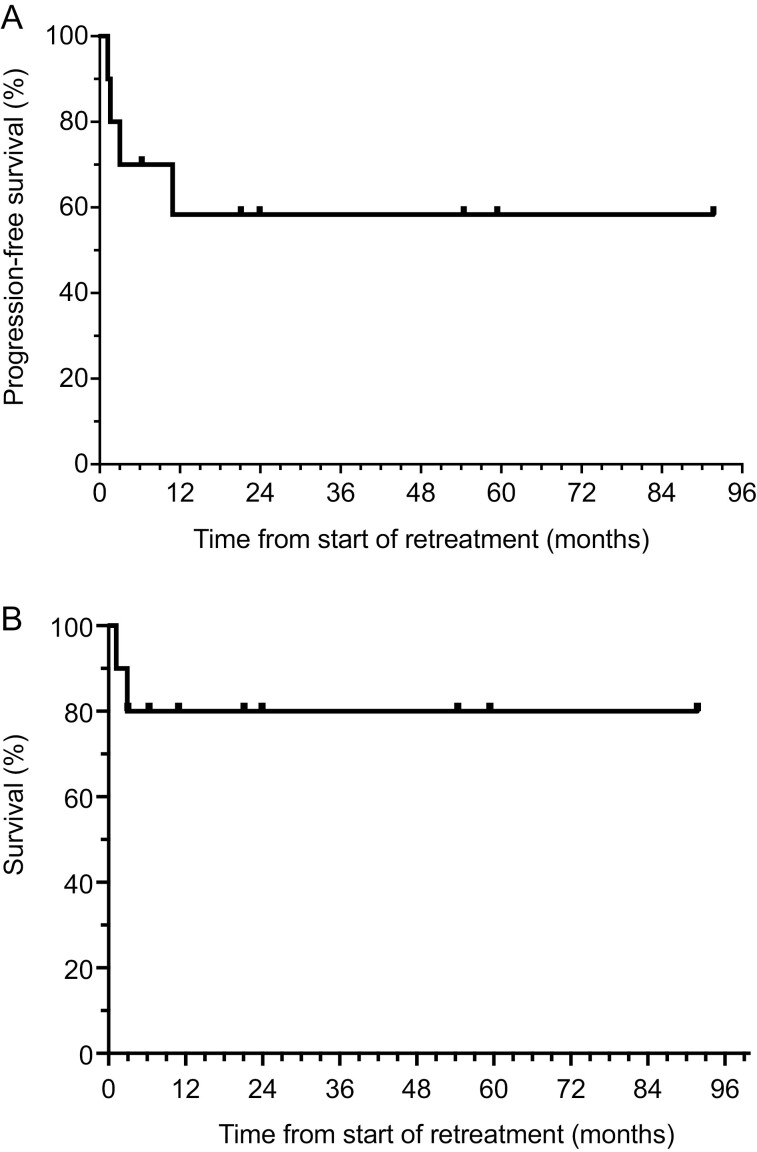
(**A**) Kaplan-Meier analysis of progression-free survival from the time of retreatment in patients who relapsed after elective treatment discontinuation (median follow-up 26.6 months). (**B**) Kaplan-Meier analysis of overall survival (OS) in patients achieving a radiologic or pathologic complete response, measured from the time of CKI re-induction.

At the time of relapse, 4 patients (40%) were found to have an elevated serum lactate dehydrogenase (LDH) level (reference range: 120-246 units/liter). If patients did not achieve a 2nd complete remission after 3 months of CKI reinduction therapy, ablation of limited residual metastatic disease with surgery or radiosurgery was considered. Patients who received local therapy all had partial responses to treatment but had persistent oligometastatic lesions despite retreatment. Five patients (50%) patients with the residual multifocal disease and a targetable mutation eventually received cautious addition of targeted therapy (BRAF ± MEK inhibitors) with continued PD-1 directed treatment, based on institutional data.^10,11^ The median PFS had not been reached with a median follow-up of 26.6 months (Fig. 1A).

With a median follow-up of 26.6 months, 6 patients (60%) currently remain in ongoing remission without evidence of recurrence. Four patients (40%) failed to respond to CKI retreatment or added targeted therapy. Three of these non-responding patients (30%) have died, while 1 continues to receive treatment. All responding patients have again discontinued therapy after a 2nd radiographically confirmed complete remission, following our institutional treatment discontinuation protocol.^4^ The median OS from the beginning of initial CKI treatment has not been reached with 70% of patients alive 2 years following reinduction therapy (Fig. 1B).

**Figure 2.**
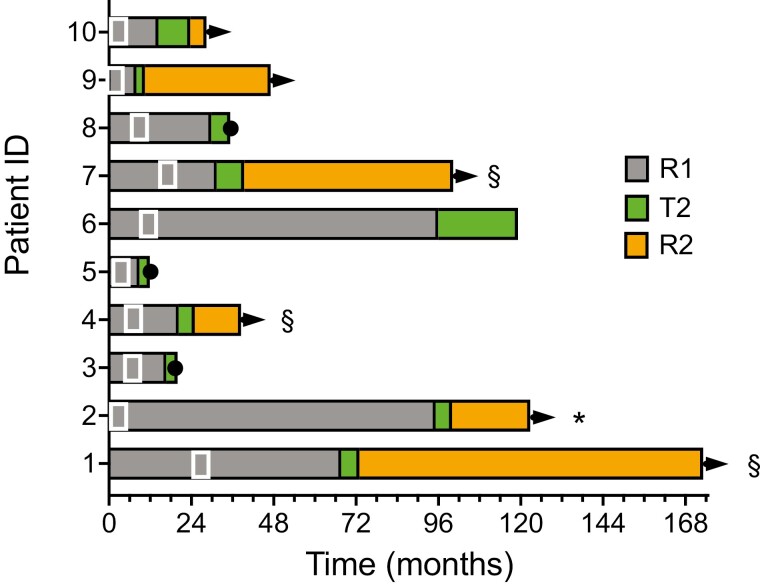
Swim-lane plot of duration of initial and 2nd line therapy. Ongoing responses are indicated by arrows. Deaths are indicated by dots at end of a line. Ongoing treatment is indicated by a blunt-ended line. The box in figure represents the time of initial CKI discontinuation. An “*” indicates addition of complete response after addition of targeted therapy, while “§” indicates patients converted to complete response by surgery or stereotactic ablative radiotherapy. R1 indicates the duration of initial complete response, T2 indicates duration of CKI retreatment, R2 indicates the duration of response following a 2nd treatment discontinuation.

The duration of initial CR and response to re-induction therapy are shown in a swim-lane plot (Fig. 2). Two patients (20%) achieved a CR with immunotherapy alone. This included patient 2 who had relapsed with a large perinephric mass and systemic fevers/chills, while patient 10 had extensive retroperitoneal axillary adenopathy. Eight patients did not achieve a 2nd CR after 3 months of CKI retreatment. Three had limited residual disease treated with surgery (*n* = 2) or radiation (*n* = 1). Two patients underwent surgical resection of their lesions, and 1 patient had a residual pulmonary metastasis ablated using cyberknife. Patients 1 and 8 did not receive additional CKI treatment after surgical resection of nodal disease and SRS ablation of residual lung metastasis. Five patients with BRAF V600E mutations had BRAF TKI added to ongoing PD-1 antibody maintenance therapy. One of these 5 also achieved a durable complete remission.

### Toxicity Related to Retreatment

Six patients (60%) experienced characteristic immune-related toxicities during CKI re-induction, including rash, pruritus, fever, vertigo, diarrhea, nausea, and vomiting (Table 1). Only 1 out of the 6 patients who experienced immune-related adverse events was forced to discontinue retreatment because of intractable toxicity, including severe vertigo, pruritus, and rash. Two other patients experienced grades 2-3 treatment-related toxicity that responded to standard management protocols (diarrhea and rash, diarrhea, and fever, respectively). There were no treatment-related hospitalizations or fatalities.

## Discussion

CKI therapy has undergone substantial evolution over recent decades. Treatment with single-agent CTLA-4 directed monoclonal antibodies has achieved 19%-25% 5-year survival.^13^ Treatment with PD-1 antibodies increased 5-year survival to the 34%-42% range and proved superior to CTLA-4 directed monotherapy.^2,14^ Combination therapy with PD-1 plus CTLA-4 antibodies has now achieved a 5-year survival of over 50%.^1^ The optimal treatment duration for CKI therapy in metastatic melanoma patients remains unknown. It is becoming clear that many completely responding patients can safely discontinue therapy. Our patient experience and that of other investigators suggest that treatment can be electively discontinued once a radiologically confirmed complete remission is achieved.^4,5,10^ Patients who achieve less than complete remission have inferior results following attempted treatment discontinuation.^4,15-17^ A small percentage of patients who electively discontinue CKI therapy appear to eventually relapse while in radiographically confirmed remission (estimated at 8.7%-10%).^4,5^

The effectiveness of re-induction using CKI therapy in relapsing patients is currently uncertain. Previously published studies suggested a 12.5%-25% 2nd CR rate (R2), although a number of these series included patients who discontinued therapy without achieving a previous CR.^2,15,18,19^ Our current case series was intended to evaluate the rare CR patients who relapse and report the response to retreatment with CKI-based therapy.

In our current series, we found that 20% of our patients were able to achieve another durable CR with CKI retreatment alone. A novel aspect of our current patient series is that the planned individualized addition of salvage treatment to PD-1-based therapy converted additional patients to CR. Treatment for these patients was individualized, based on the characteristics of their relapse. If the relapse was oligometastatic, ablation of the disease with surgery or radiosurgery was considered. In the case of a multiorgan pattern of relapse, patients were evaluated for the presence of targetable tumor DNA mutations (eg, BRAF, NRAS, and NF1). If a targetable mutation was identified, cautious addition of BRAF or MEK inhibitor therapy to ongoing PD-1 maintenance therapy was used.^10,11^ This individualized approach allowed 4 additional patients (40%) to achieve a 2nd complete remission.

We conclude CKI retreatment is useful as an initial salvage option in patients who relapse after initial CKI-induced complete remissions. Continued CKI treatment can be successfully combined with other modalities, such as surgery, radiotherapy, or targeted agents, to allow additional patients to be converted to durable CR.

It should be noted that the time to relapse following initial CKI treatment (R1) was a median of 25 months. In our series, patients with a shorter interval to relapse (ie, initial remission/stable disease <24 months following initial CKI induction) had a similar outcome with retreatment as patients who progressed after 24 months. Interestingly, the 2 patients responding to CKI re-induction alone relapsed at less than 24 months following initial CKI induction. Also, no patients achieving a 2nd remission have progressed after 24 months, despite a 2nd elective treatment discontinuation. LDH, PD-L1 expression, and tumor mutational burden did not appear to correlate with 2nd responses, but this patient series is too small for meaningful statistical analysis. Future studies should continue to explore the relationship between potential biomarkers and the efficacy of CKI retreatment in metastatic melanoma patients. Another area of future research revolves around a better understanding of the pathophysiology driving resistance to CKI retreatment in relapsing patients.

As increasing numbers of CKI-treated patients achieve complete remissions, a small percentage will eventually relapse. Our case series is meant to encourage the development of prospective clinical trials to address this developing clinical challenge. Potential limitations of the present study include that it represents a retrospective chart review encompassing 14 years of patient data. The patients in our study were treated with a variety of CKI agents available during induction and re-induction because of changes in treatment paradigms over this lengthy period.

## Conclusions

Patients with metastatic melanoma who achieve a radiologically confirmed complete remission have a low likelihood of relapse after elective treatment discontinuation. Eventual relapse in a small percentage of these patients is a developing clinical challenge. In this unique population, CKI retreatment alone induces a durable 2nd remission in approximately 20% of patients. Additional patients can be converted to durable CR by individualized addition of potential salvage therapies to continued PD-1 maintenance therapy. Further evaluation of treatment options for this challenging patient population is needed.

## Data Availability

The data underlying this article will be shared on reasonable request to the corresponding author.

## References

[1] Wolchok JD , Chiarion-Sileni V, Gonzalez R, et al. Long-term outcomes with nivolumab plus ipilimumab or nivolumab alone versus ipilimumab in patients with advanced melanoma. J Clin Oncol. 2022;40:127-137. 10.1200/JCO.21.02229 34818112 PMC8718224

[2] Hamid O , Robert C, Daud A, et al. Five-year survival outcomes for patients with advanced melanoma treated with pembrolizumab in KEYNOTE-001. Ann Oncol. 2019;30:582-588. 10.1093/annonc/mdz011 30715153 PMC6503622

[3] Larkin J , Chiarion-Sileni V, Gonzalez R, et al. Five-year survival with combined nivolumab and ipilimumab in advanced melanoma. N Engl J Med. 2019;381:1535-1546. 10.1056/NEJMoa1910836 31562797

[4] Perez L , Samlowski W, Lopez-Flores R. Outcome of elective ­checkpoint inhibitor discontinuation in patients with metastatic melanoma who achieved a complete remission: real-world data. Biomedicines. 2022;10(5):1144-1159. 35625881 10.3390/biomedicines10051144 PMC9138966

[5] Robert C , Ribas A, Hamid O, et al. Durable complete response after discontinuation of pembrolizumab in patients with metastatic melanoma. J Clin Oncol. 2018;36:1668-1674. 10.1200/JCO.2017.75.6270 29283791

[6] U.S. Department of Health and Human Services NIoH, National Cancer Institute. *Common Terminology Criteria for Adverse Events (CTCAE) Version 4.0*, 2010.

[7] Thompson JA , Schneider BJ, Brahmer J, et al. Management of immunotherapy-related toxicities, version 1.2022, NCCN clinical practice guidelines in oncology. J Natl Compr Canc Netw. 2022;20:387-405. 10.6004/jnccn.2022.0020 35390769

[8] Eisenhauer EA , Therasse P, Bogaerts J, et al. New response evaluation criteria in solid tumours: revised RECIST guideline (version 1.1). Eur J Cancer. 2009;45:228-247. 10.1016/j.ejca.2008.10.026 19097774

[9] Lebbe C , Meyer N, Mortier L, et al. Evaluation of two dosing regimens for nivolumab in combination with ipilimumab in patients with advanced melanoma: results from the Phase IIIb/IV CheckMate 511 trial. J Clin Oncol. 2019;37:867-875. 10.1200/JCO.18.01998 30811280 PMC6455714

[10] Samlowski W , Adajar C. Cautious addition of targeted therapy to PD-1 inhibitors after initial progression of BRAF mutant metastatic melanoma on checkpoint inhibitor therapy. BMC Cancer. 2021;21:1187-1199. 10.1186/s12885-021-08906-1 34743688 PMC8573907

[11] Hilts A , Samlowski W. Cautious addition of MEK inhibitors to PD-1 antibody treatment in patients with NRAS or NF1 mutant metastatic melanoma failing initial immunotherapy. Ann Case Rep. 2022;7:795-805. 10.29011/2574-7754.100795

[12] Kaplan EL , Meier P. Nonparametric estimation from incomplete observations. J Am Stat Assoc. 1958;53:457-481. 10.1080/01621459.1958.10501452

[13] Ascierto PA , Del Vecchio M, Mackiewicz A, et al. Overall survival at 5 years of follow-up in a phase III trial comparing ipilimumab 10 mg/kg with 3 mg/kg in patients with advanced melanoma. J Immunother Cancer. 2020;8. 10.1136/jitc-2019-000391 PMC7279645 32503946

[14] Atkins MB , Lee SJ, Chmielowski B, et al. DREAMseq (doublet, randomized evaluation in advanced melanoma sequencing): a phase III trial—ECOG-ACRIN EA6134. J Clin Oncol. 2021;39:356154-356154. 10.1200/JCO.2021.39.36_suppl.356154

[15] Betof Warner A , Palmer JS, Shoushtari AN, et al. Long-term outcomes and responses to retreatment in patients with melanoma treated with PD-1 blockade. J Clin Oncol. 2020;38:1655-1663. 10.1200/JCO.19.01464 32053428 PMC7238490

[16] Pokorny R , McPherson JP, Haaland B, et al. Real-world experience with elective discontinuation of PD-1 inhibitors at 1 year in patients with metastatic melanoma. J Immunother Cancer. 2021;9. 10.1136/jitc-2020-001781 PMC7843310 33500258

[17] Asher N , Israeli-Weller N, Shapira-Frommer R, et al. Immunotherapy discontinuation in metastatic melanoma: lessons from real-life clinical experience. Cancers. 2021;13. 10.3390/cancers13123074 PMC8234591 34203061

[18] Jansen YJL , Rozeman EA, Mason R, et al. Discontinuation of ­anti-PD-1 antibody therapy in the absence of disease progression or treatment limiting toxicity: clinical outcomes in advanced melanoma. Ann Oncol. 2019;30:1154-1161. 10.1093/annonc/mdz110 30923820

[19] Robert C , Marabelle A, Herrscher H, et al. Immunotherapy discontinuation—how, and when? Data from melanoma as a paradigm. Nat Rev Clin Oncol. 2020;17:707-715. 10.1038/s41571-020-0399-6 32636502

